# Dentistry and Intensive Care Unit: A Brief Report

**DOI:** 10.1055/s-0041-1735797

**Published:** 2021-12-01

**Authors:** Lisiane Cristina Bannwart, Clóvis Lamartine de Moraes Melo Neto, Daniela Micheline dos Santos, André Luiz de Melo Moreno, Aldiéris Alves Pesqueira, Marcelo Coelho Goiato, André Pinheiro de Magalhães Bertoz

**Affiliations:** 1 Department of Dental Materials and Prosthodontics, School of Dentistry, São Paulo State University, Aracatuba, São Paulo, Brazil; 2 Oral Oncology Center, School of Dentistry, São Paulo State University, Araçatuba, São Paulo, Brazil; 3 Department of Pediatric and Social Dentistry, School of Dentistry, São Paulo State University, Araçatuba, São Paulo, Brazil

**Keywords:** complete denture, intensive care units, removable partial denture, dental prosthesis, cross infection, acrylic resin

## Abstract

**Objective** The aim of this study is to verify whether removable dentures of patients admitted to an intensive care unit (ICU) are niches of microorganisms that can cause pathologies ( *Staphylococcus aureus* , *Candida spp.* , and enterobacteria).

**Materials and Methods** Fifteen patients who were denture wearers (removable partial denture and complete denture) were included in this study. Patients must wear their dentures daily, and these dentures must have acrylic parts. Microbial biofilm was collected from the acrylic part of one denture of each patient. Then, the biofilm was seeded on different culture media: Sabouraud agar, blood agar, MacConkey agar, and mannitol salt agar. In this study, biochemical evaluations of microorganisms were performed.

**Statistical analysis** The percentage of dentures with the microorganism identified by each culture medium was calculated.

**Results** In total, 100% of the dentures were positive for *Staphylococcus spp.* (blood agar) and *Candida spp.* (Sabouraud agar); 33.3% of the dentures were positive for *S. aureus* (Mannitol salt agar); and 13.3% of the dentures were positive for *Shigella spp.* (MacConkey agar).

**Conclusion** Removable dentures of patients (removable partial dentures and complete dentures) admitted to an ICU are niches of microorganisms that can cause pathologies.

## Introduction

Intensive care units (ICUs) have contributed to the survival of patients with trauma and other life-threatening conditions. 1 Despite this, ICUs are associated with a high risk of nosocomial infections. 1 2

Nosocomial infections are infections that were not present in the patient at the time of admission to a health facility and appear 48 hours after the patient's hospital admission. 3 4 It is estimated that the incidence of these infections is two to five times higher in ICUs than in the general hospitalized population. 2 According to Ak et al, ICU patients are at increased risk of developing nosocomial infections for a variety of reasons, including presence of underlying diseases, prolonged ICU stay, invasive diagnostic and monitoring procedures performed, impaired host defenses, and colonization by resistant microorganisms. 2

Nosocomial infections can affect any part of the body; however, the respiratory tract is the region most often affected (nosocomial pneumonia). 2 5 Nosocomial pneumonia can occur when the patient aspirates microorganisms (respiratory pathogens), and these microorganisms reach the lower respiratory tract. 6 7 Microbial plaque can be a source of respiratory pathogens, and it can be found on teeth and removable dentures. 6 7

Based on the fact that elderly patients admitted to an ICU may be wearers of removable dentures (removable partial denture and complete denture), 8 and that aging is associated with an increased risk of hospital-acquired infections 9 ; the aim of this study is to verify whether removable dentures of patients admitted to an ICU are niches of microorganisms that can cause pathologies ( *S. aureus* , *Candida spp.* , and enterobacteria).

## Materials and Methods

### Ethics Committee

This study was approved by the ethics committee of the Paulista University (number: 45973115.9.0000.5512). This study included patients admitted to the ICU of the Hospital 28 de Agosto in the city of Manaus (Brazil). This study was conducted between September and December 2017.

Initially, 68 patients admitted to the ICU were selected. After applying the inclusion and exclusion criteria, 15 patients (11 men and 4 women) were included in this study.

### Inclusion Criteria

- Men and women over 18 years of age.
- Each patient must wear at least one removable denture (complete denture or removable partial denture).
- Removable dentures must have acrylic parts.
- Patients must wear their removable dentures in the ICU.
- Patients must remain in the ICU for at least 6 days.
- Patients must wear their removable dentures daily.
- No use of antibiotics.

### Exclusion Criteria

- Patients with bronchospasm, altered intracranial pressure, or hemodynamic instability.
- Patients in terminal stage.
- Patients who refused to participate in this study.

### Sample Collection

The team of nurses performed the cleaning of the oral tissue and removable dentures of patients. The patients' oral tissue was cleaned with a sterile gauze moistened with 0.12% chlorhexidine. Removable dentures were cleaned with sterile water and sterile gauze. There were no dentists working at the hospital.

After cleaning one denture of each patient, the biofilm was collected on the acrylic part of the denture. A sterile cotton swab moistened with sterile saline was rubbed over the acrylic base of the denture for 1 minute. After biofilm collection, the swab was placed in a test tube (16 × 160 mm) with 3 mL of sterile buffered saline solution (phosphate buffered saline), and this set was stored in a thermobox at a temperature between 2°C and 8°C. Then, the collected material was sent to the microbiology laboratory of the Paulista University.

The biofilm was seeded on different culture media: Sabouraud agar, blood agar, MacConkey agar, and Mannitol salt agar. Petri dishes were placed in a laboratory oven at 35°C, and after 48 hours, the presence or absence of colonies was checked. In this study, biochemical evaluations of microorganisms were performed.

The percentage of dentures with the microorganism identified by each culture medium was calculated.

## Results

The mean age of the participants was approximately 71 years, and the mean time of wearing the same denture or pair of dentures was 10.5 years.

All partially edentulous patients had dental calculus and gingival inflammation. In addition, in 100% of patients the following characteristics were observed: (1) removable dentures in poor condition (bacterial calculus adhesion on dentures, wear of acrylic teeth, and loss of retention and stability of dentures); (2) patients slept with their dentures removable; (3) tongue coating; and (4) oral tissue with reddened areas, suggesting the presence of candidiasis.

Table 1 shows that 100% of the dentures were positive for *Staphylococcus spp.* (blood agar) and *Candida spp.* (Sabouraud agar). It was also observed that 33.3% of the dentures were positive for *S. aureus* (Mannitol salt agar); and 13.3% of dentures were positive for *Shigella spp.* (MacConkey agar).

**Table 1:** Results of different culture media

Sex		Denture type	Nutrition	Primary disease	Blood Agar	Sabouraud Agar	Mannitol Salt Agar	MacConkey Agar	Systemic complications
1	Male a	Upper RPD	Parenteral	Cardiorespiratory arrest	Positive	*Candida spp.*	*Staphylococcus* spp.	Negative	
2	Male a	Upper CD	Oral	Cerebral vascular disease	Positive	*Candida spp.*	*Staphylococcus* spp.	Negative	Respiratory insufficiency
3	Male a	Upper CD	Oral	Acute congestive heart failure and respiratory failure	Positive	*Candida spp.*	*Staphylococcus* spp.	Negative	Pneumonia
4	Female	Upper and lower CD	Oral	Diabetic ketoacidosis	Positive	*Candida spp.*	*Staphylococcus aureus*	Negative	Pneumonia/death
5	Male a	Upper CD	Parenteral	Acute myocardial infarction	Positive	*Candida spp.*	*Staphylococcus* spp.	Negative	
6	Male	Upper and lower RPD	Oral	Unstable angina	Positive	*Candida spp.*	*Staphylococcus aureus*	Negative	Pneumonia
7	Male	Upper and lower RPD	Oral	Cardiac tamponade	Positive	*Candida spp.*	*Staphylococcus* spp.	Negative	Respiratory insufficiency/death
8	Female a	Upper RPD	Oral	Hypophosphatemia and muscle weakness	Positive	*Candida spp.*	*Staphylococcus* spp.	Positive	Diarrhea and death
9	Female	Upper and lower CD	Parenteral	Severe acidosis	Positive	*Candida spp.*	*Staphylococcus* spp.	Negative	
10	Female	Upper and lower CD	Oral	Acute myocardial infarction	Positive	*Candida spp.*	*Staphylococcus* spp.	negative	Diarrhea
11	Male	Upper and lower CD	Oral	Acute myocardial infarction	Positive	*Candida spp.*	*Staphylococcus aureus*	negative	Pneumonia
12	Male	Upper and lower CD	Oral	Diabetic ketoacidosis	Positive	*Candida spp.*	*Staphylococcus aureus*	negative	Pneumonia
13	Male	Upper and lower CD	Oral	Acute myocardial infarction	Positive	*Candida spp.*	*Staphylococcus* spp.	Positive	Diarrhea
14	Male	Upper and lower CD	Oral	Diabetic ketoacidosis	Positive	*Candida spp.*	*Staphylococcus* spp.	Negative	
15	Male	Upper and lower CD	Oral	Cardiorespiratory arrest	Positive	*Candida spp.*	*Staphylococcus aureus*	Negative	Pneumonia
Total	66.6% Male				100% Positive	100% *Candida spp* .	66.7% *Staphylococcus* spp.	86.7% Negative	
	33.4% Female				0% Negative		33.3% *Staphylococcus aureus*	13.3% Positive	

Abbreviations: CD, complete denture; RPD, removable partial denture.

a Presence of natural teeth in the lower dental arch.

## Discussion

In the present study, teeth and mucosa were cleaned with 0.12% chlorhexidine. Chlorhexidine can destroy bacteria by breaking their membranes. 10 Another method that can be used in conjunction with chemical cleaning is mechanical brushing of the teeth, mucosa and tongue. Thus, for ICU patients, it is important that brushing (mechanical cleaning) be performed before chemical cleaning (0.12% chlorhexidine) to remove the tongue coating, bacterial plaque and food remains. 8 In the present study, tongue coating, dental calculus, gingival inflammation and oral tissue with reddened areas were observed in patients, therefore, the presence of a dentist in an ICU could help to prevent or control these situations. According to Miranda et al, biofilm on tooth surfaces, coated tongues, and periodontal disease tend to aggravate patient clinical conditions because they offer an optimal environment for growth of Gram-negative bacteria. 8 This growth results in more virulent oral microflora. 8

In this study, it was possible to identify pathogens on removable dentures ( Table 1 ). The poor hygiene of a removable denture allows the accumulation of biofilm on it, and this can contribute to the development of several oral and systemic infections. 10 Thus, cleaning removable dentures is important to avoid these problems. 10 Literature reports that brushing removable dentures with soap, associated with chemical disinfection (e.g., 2% chlorhexidine) is effective in reducing the colonization of microorganisms ( *Candida spp* . and *S. aureus* ) on them. 11 12 13 14 15

Patients evaluated in this study slept with their dentures removable. Individuals who sleep with their dentures removable tend to have poor oral hygiene practices, high rates of microbial plaque on their tongues and dentures, oral candidiasis, and visit their dentists less often. 16 Thus, these individuals may have an increased risk of aspirating microorganisms that can cause pneumonia. 16 Therefore, this is particularly dangerous for patients admitted to an ICU because their immunity is usually reduced. 2 This may explain the pneumonia events observed in Table 1 .

Removable dentures (removable partial dentures and complete dentures) should normally be replaced after 5 years of constant use. 17 18 Over time, the roughness of removable dentures can increase due to their cleaning and daily use, favoring the adhesion of microorganisms. 14 19 In this study, the mean time of wearing the same denture or pair of dentures was 10.5 years. This suggests that the surface roughness of the evaluated dentures was high.

Patients aged 65 and over are more prone to hospital infections due to generally low immunity, impaired physiological function, chronic illness, and medication use (e.g., immunosuppressants, anticholinergics, sedatives, and gastric acid inhibitors). 9 Therefore, these patients have a high mortality rate. 9 In the present study, the mean age of patients was approximately 71 years. 9 Therefore, advanced age of the evaluated patients was a risk factor.

The most frequent etiologic agents that cause hospital infections are *S. aureus* , *Klebsiella pneumoniae* , *Pseudomonas aeruginosa* , *Acinetobacter spp.* , *Escherichia coli* , *Enterobacter spp* ., and *Candida spp* . 20 21 22 In this study, two of these microorganisms ( *S. aureus* and *Candida spp.* ) were identified on removable dentures of patients included in this study ( Table 1 ).

*Staphylococci aureus* are responsible for skin and soft tissue infections, surgical site infections, endocarditis, pneumonia, and hospital-acquired bacteremia. 23 24 In this study, 33.3% of the dentures were positive for *S. aureus* (Mannitol salt agar). Coincidentally, the wearers of these removable dentures had nosocomial pneumonia ( Table 1 ). Thus, it is possible that they have aspirated this microorganism causing this systemic complication. 8

In the ICUs, *Candida spp* . may be associated with nosocomial infection, 25 morbidity, and mortality. 26 All dentures evaluated in this study were positive for *Candida spp.* It is worth noting that *Candida albicans* is the most common species found in the mouth. 25 27 According to Akpan et al, the wearing of dentures produces a microenvironment with low oxygen and low pH, and this favors the growth of *Candida spp* . 27 In addition, the ease of adhesion of *Candida spp.* to acrylic, reduced flow of saliva under the surfaces of the dentures, and lack of oral hygiene can contribute to the development of candidiasis. 27 Thus, all of this information may explain the presence of *Candida spp.* in all samples evaluated ( Table 1 ).

According to Formal et al, invasive pathogens such as the *Shigella spp* . damage the intestinal mucosa. 28 This mucosal damage, which may range from a mild inflammatory response to gross ulceration, is a consequence of this pathogen's ability to invade the intestinal epithelium. 28 In this study, 13.3% of dentures were positive for *Shigella spp* ., and their wearers have had diarrhea (systemic complication). Therefore, it is possible that this pathogen caused this systemic complication.

Thus, based on the present study, the dentist is extremely important in an ICU.

## Conclusion

Removable dentures of patients (removable partial dentures and complete dentures) admitted to an ICU are niches of microorganisms that can cause pathologies.
